# Effect of Alkyl Side Chain Length on Electrical Performance of Ion-Gel-Gated OFETs Based on Difluorobenzothiadiazole-Based D-A Copolymers

**DOI:** 10.3390/polym16233287

**Published:** 2024-11-26

**Authors:** Han Zhou, Zaitian Cheng, Guoxing Pan, Lin Hu, Fapei Zhang

**Affiliations:** 1 Anhui Key Laboratory of Low-Energy Quantum Materials and Devices, High Magnetic Field Laboratory, HFIPS, Chinese Academy of Sciences, Hefei 230031, China; hzhouhmfl@163.com (H.Z.); ustckxdczt@163.com (Z.C.); hulin@hmfl.ac.cn (L.H.); 2 Science lsland Branch, Graduate School, University of Science and Technology of China, Hefei 230026, China; 3 Institutes of Physical Science and Information Technology, Anhui University, Hefei 230601, China

**Keywords:** D-A copolymer, organic semiconductor, organic field effect transistors, ion-gel dielectric, flexible electronic

## Abstract

The performance of organic field-effect transistors (OFETs) is highly dependent on the dielectric–semiconductor interface, especially in ion-gel-gated OFETs, where a significantly high carrier density is induced at the interface at a low gate voltage. This study investigates how altering the alkyl side chain length of donor–acceptor (D-A) copolymers impacts the electrical performance of ion-gel-gated OFETs. Two difluorobenzothiadiazole-based D-A copolymers, PffBT4T-2OD and PffBT4T-2DT, are compared, where the latter features longer alkyl side chains. Although PffBT4T-2DT shows a 2.4-fold enhancement of charge mobility in the SiO_2_-gated OFETs compared to its counterpart due to higher crystallinity in the film, PffBT4T-2OD outperforms PffBT4T-2DT in the ion-gel-gated OFETs, manifested by an extraordinarily high mobility of 17.7 cm^2^/V s. The smoother surface morphology, as well as stronger interfacial interaction between the ion-gel dielectric and PffBT4T-2OD, enhances interfacial charge accumulation, which leads to higher mobility. Furthermore, PffBT4T-2OD is blended with a polymeric elastomer SEBS to achieve ion-gel-gated flexible OFETs. The blend devices exhibit high mobility of 8.6 cm^2^/V s and high stretchability, retaining 45% of initial mobility under 100% tensile strain. This study demonstrates the importance of optimizing the chain structure of polymer semiconductors and the semiconductor–dielectric interface to develop low-voltage and high-performance flexible OFETs for wearable electronics applications.

## 1. Introduction

Flexible electronics enable light-weight and skin-like devices based on stretchable films, paving the way for innovative applications in wearables, healthcare, and smart textiles [1,2,3,4]. Organic field effect transistors (OFETs), attributed to the ease of large-area solution preparation, intrinsic flexibility, and light-weight polymer semiconductors, are attractive materials used for electronic skins, conformable bio-sensors, and stretchable integrated circuits [5,6,7,8,9,10,11].

Nevertheless, the rigid backbones and high crystallinity of polymer semiconductors, while facilitating carrier transport, also limit their application in flexible electronic devices. There are two primary methods developed to improve the mechanical flexibility of polymer semiconductors: (1) backbone design and side-chain engineering, by which flexible non-conjugated group units are incorporated or dynamic bonding is introduced to enhance dynamic interchain interactions [12,13]; (2) blending with insulating elastomers, where stretchable elastomeric polymers, such as PDMS or SEBS, are physically blended with the semiconductors to enhance the flexibility of the semiconducting films [14,15,16]. Compared to the complex chemical synthesis required for molecular structural engineering, the blending strategy is a relatively straightforward process [17,18], which offers a versatile and accessible route for enhancing flexibility in a wide variety of organic semiconductors. For instance, Zhang et al. demonstrated a blend of PDMS with the semiconducting polymer poly(3-hexylthiophene) (P3HT) to fabricate flexible OFET arrays [15]. The devices exhibited excellent mechanical flexibility, retaining more than 90% of their electrical performance after 300 stretching cycles at 100% strain. Xu et al. introduced a block copolymer elastomer SEBS to blend with a diketopyrrolopyrrole-based (PDPP-TT) donor–acceptor (D-A) polymer to produce highly stretchable polymer semiconductor films [19]. This study demonstrated that the SEBS/PDPP-TT blends produced devices with high strain tolerance and mobility exceeding 1.0 cm^2^/V s at 100% strain, attributed to the nanoconfinement effect in the blend matrix.

Recently, soft and flexible ion-gel dielectrics, which consist of a polymer matrix infused with ionic liquids, have gained significant attention for the fabrication of wearable devices [20,21,22,23]. Additionally, flexible OFETs with ion-gel dielectrics can operate at much lower voltages (typically below 5 V) due to the formation of an electric double layer (EDL) [24,25,26,27,28], making them ideal for next-generation flexible electronics and wearable devices. For example, Shim et al. introduced a stretchable synaptic transistor based on ion-gel dielectrics, mimicking biological synapses, for use in flexible, neurologically integrated devices such as sensory skins and soft robots [29]. Chen et al. introduced a directly written ion-gel dielectric layer in flexible OFETs, demonstrating the significance of ion-gel dielectrics in high-performance flexible OFETs for health monitoring and signal amplification [30]. Specifically, ion–electronic interfacial coupling, closely associated with the molecular structure of polymers, ion-gel electrolytes, and polymer stacking, is critical to the performance of ion-gel-gated OFETs [31,32,33,34]. Among these factors, the molecular structure of polymers is a key determinant of carrier transport and ion penetration at the polymer/ion-gel interface [35]. Therefore, optimizing the backbone and side chains of polymer semiconductors is crucial for enhancing the performance of ion-gel-gated OFETs, particularly in the development of wearable and flexible electronics. However, research on ion-gel dielectric-gated OFETs using D-A copolymers, which exhibit excellent carrier transport capability due to the high coplanarity of their backbones [36,37,38], remains scarce [39,40,41]. Furthermore, the effects of the microstructures and the semiconductor/EDL interface properties of the D-A copolymers on the OFET performance are far from being understood so far.

**Figure 1:**
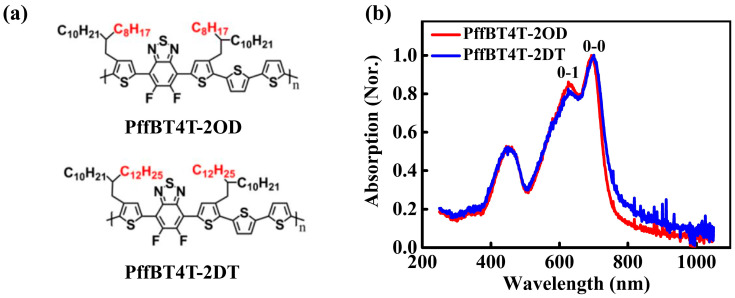
(**a**) Chemical structure of PffBT4T-2OD and PffBT4T-2DT. (**b**) The normalized UV-visible absorption spectra of the PffBT4T-2OD and PffBT4T-2DT film.

In this study, we focus on two difluorobenzothiadiazole-based D-A copolymers (denoted as PffBT4T-2OD and PffBT4T-2DT, respectively, in Figure 1a) with strong interchain aggregation [42,43,44,45]. Detailed examinations are made on the influence of the alkyl side chain length of the polymers on electrical performance in both SiO_2_-gated and ion-gel-gated OFETs. Microstructural characterizations are employed to evaluate the crystallinity and molecular packing of the polymer films. In SiO_2_-gated OFETs, PffBT4T-2DT shows superior crystallinity and enhanced carrier mobility compared to PffBT4T-2OD, attributed to its longer alkyl side chains, which results in enhanced π-π stacking and larger crystalline domains. Conversely, in ion-gel-gated OFETs, PffBT4T-2OD outperforms PffBT4T-2DT due to its smoother surface morphology as well as stronger interaction with the ion-gel dielectric. Furthermore, PffBT4T-2OD is blended with the elastomer SEBS to construct stretchable low-voltage OFETs. The ion-gel-gated PffBT4T-2OD/SEBS blend devices show superior mobility of 8.6 cm^2^/V s, as well as high mechanical flexibility, retaining 45% of the initial FET mobility under 100% tensile strain. This study offers new insights into the role of the chain structure of polymeric semiconductors in the performance of ion-gel-gated OFETs. The findings also emphasize the importance of optimizing the semiconductor–dielectric interface to achieve high-performance, flexible OFETs.

## 2. Materials and Methods

### 2.1. Materials

The PffBT4T-2OD (Mw: 30 kDa, PDI: 2.2) and PffBT4T-2DT (Mw: 30 kDa, PDI: 2.2) were purchased from Solarmer Materials Inc (Beijing, China). Ion liquid [EMIM]^+^[TFSI]^−^ (1-Ethyl-3-methylimidazolium bis(trifluoromethylsulfonyl)imide, ≥97%) and P(VDF-HFP) were purchased from Merk Co., Ltd. (Darmstadt, Germany). Acetone and 1,2-Dichlorobenzene (o-DCB, 99%) were purchased from Aladdin Biochemical Technology Co., Ltd. (Shanghai, China). All materials were used as received.

### 2.2. Structural Characterisation

UV-vis-NIR absorption spectra were measured using a Shimadzu UV-3100 spectrophotometer from Shimadzu Inc. (Kyoto, Japan) with transmission geometry for the samples deposited on quartz substrates. Two-dimensional (2D) grazing incidence X-ray diffraction (GIXRD) measurements were conducted at the beamline BL14B of the Shanghai Synchrotron Radiation Facility (SSRF) (Shanghai, China) with a photon energy of 10.0 keV. The incidence angle of the X-ray beam was 0.2°. The distortion correction was made before performing the quantitative analysis of the 2D GIXRD images. The surface morphologies of the films were probed using a Veeco MultiMode (Nanoscope V) atomic force microscope (AFM) from Bruker Inc. (Berlin, Germany) in tapping mode.

### 2.3. The Preparation of Ion-Gel Based Capacitor

A solution of ion-gel was prepared by dissolving P(VDF-HFP) and [EMIM]^+^[TFSI]^−^ in acetone with mass ratio of 1:4:7. Then, the solution was stirred at 40 °C for 6h. Ion-gel film was spun from the ion-gel solution on a clean Si/SiO_2_ substrate at a speed of 500 rpm for 60 s and then annealed at 70 °C for 1 h. The film thickness was measured as ca. 0.5 mm. A strip of Cr/Au (5 nm/80 nm) with a width of 0.2 cm and a length of 1.5 cm was vapor deposited on the clean SiO_2_/Si substrate using shadow mask as bottom electrode. The ion-gel films with a size of 0.3 × 0.3 cm^2^ was then cut and laminated on the deposited Au electrode. Finally, a 100 nm Au stripe with a size of 0.2 cm × 1.5 cm, pre-deposited on an elastomer PDMS substrate, was transferred and attached to the ion-gel film as top electrode to form a cross-bar structure with an effective area of 0.2 × 0.2 cm^2^.

### 2.4. The Preparation of Ion-Gel-Gated OFETs

PffBT4T-2OD and PffBT4T-2DT were dissolved in o-dichlorobenzene. The solutions (6 mg/mL) were stirred at 80 °C /12 h for complete dissolution in a N_2_ glove box, and then solution was spun-coated at a speed of 1000 rpm for 60 s on a clean Si/SiO_2_ substrate pre-patterned with the Cr/Au (5 nm/80 nm) source/drain electrode arrays using shadow mask. The electrode arrays had a channel width (W) of 2.0 mm and channel length (L) of 50 µm, 70 µm, 100 µm, and 200 µm, respectively. Then, the films were annealed at 140 °C for 1 h under nitrogen atmosphere to remove the residual solvent. Finally, the ion-gel film was cut and laminated on the channel region as dielectric, and a 100 nm Au layer was transferred and attached on the ion-gel film as gate electrode to fabricate top-gate/bottom-contact (TG/BC) ion-gel-gated OFET devices.

### 2.5. Electrical Properties’ Characterization

The electrical characteristics of the ion-gel-gated OFETs were measured on a probe station using a Keithley 2612A source meter (Tektronix, Inc., Beaverton, OR, USA) in nitrogen atmosphere. Carrier mobility (μ) was calculated from the slope of the square root of drain current (I_D_) versus the gate voltage (V_G_) curves in a saturated regime according to the transistor equation as follows [20,46]:

$$\mu =\frac{L}{WC_{i}}{\left(\frac{\partial \sqrt{I_{D}}}{\partial V_{G}}\right)}^{2}$$

where Ci is the area capacitance of the ion-gel dielectric at 10 Hz. The frequency dependence of the capacitance and electrochemical impedance spectroscopy (EIS) was measured using an impedance analyzer (Agilent 4294A from Agilent Technologies, Inc. in Santa Clara, CA, USA) as well as an electrochemical workstation (Zennium, Inc. in Kronach, Germany), respectively.

## 3. Results and Discussion

### 3.1. Microstructures of PffBT4T-2OD and PffBT4T-2DT Films

The polymer PffBT4T-2DT possesses longer alkyl side chains compared with its homologue PffBT4T-2OD. UV-visible absorption spectroscopy is used to investigate the effect of alkyl side chain length on the inter-chain aggregation behavior of D-A copolymers. The UV-visible absorption spectra of the PffBT4T-2OD and PffBT4T-2DT films are shown in Figure 1b. Strong vibrational peaks around 700 nm and 630 nm, assigned to the 0-0 and 0-1 transitions [45,47], are observed on both samples. This indicates that both polymers exhibit strong aggregation in the film state. Strong inter-chain aggregation behavior can also be found in the solutions of both polymers, as shown in Figure S1 of the Supporting Information. Although both of the films display similar absorption behavior, the A_0-0_/A_0-1_ ratio (where A_0-0_ and A_0-1_ represent the relative intensities of the 0-0 and 0-1 peaks, respectively) is larger for the PffBT4T-2DT film. Moreover, the 0-0 peak position of the PffBT4T-2DT film is slightly red-shifted compared to that of the PffBT4T-2OD film. It is suggested that PffBT4T-2DT films, with longer alkyl side chains, exhibit stronger inter-chain interactions [48,49].

**Figure 2:**
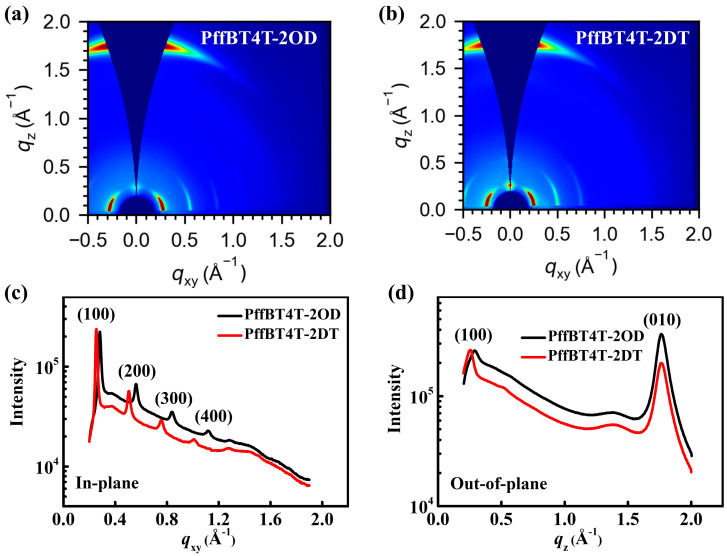
(**a**,**b**) Two-dimensional GIXRD patterns of the PffBT4T-2OD film (**a**) and PffBT4T-2DT film (**b**), respectively; (**c**,**d**) Cross-section profiles along the *q_xy_* (**c**) and *q_z_* (**d**) directions of the GIXRD patterns shown in (**a**,**b**).

Grazing incidence X-ray diffraction (GIXRD), a powerful and precise technique for characterizing molecular packing order in the crystalline regions of polymer films, is employed to investigate the effect of alkyl side chain length on the molecular packing of D-A copolymers. The 2D-GIXRD patterns of both PffBT4T-2OD and PffBT4T-2DT films are shown in Figure 2a,b. Both the films exhibit a strong and broad π-π stacking (010) diffraction peak around *q_z_* = 1.76 Å^−1^ along the out-of-plane scattering vector *q_z_*, indicating the face-on chain packing order [50]. Moreover, the corresponding cross-section profiles along the *q_xy_* and *q_z_* direction are shown in Figure 2c and Figure 2d, respectively. Four distinct (h00) series reflections, assigned to lamellar stacking, are observed along the in-plane direction (*q_xy_)* for both samples. This thus confirms that both PffBT4T-2OD and PffBT4T-2DT films exhibit long-range and highly ordered molecular stacking, which is favorable to facilitate interchain carrier transport. To better understand the microstructure of both kinds of the films, the full width at half maximum (FWHM) of the (010) and (100) peaks (shown in Figure S1, Supporting Information) were measured to calculate the crystalline correlation length (CCL) based on the Scherrer equation (as described in Note 2 of the Supporting Information). Additionally, d-spacing for the interchain packing (a detailed calculation is illustrated in Note 2 of the SI), FWHM, and CCL are summarized for two polymers in Table 1. The table indicates that the π-π stacking distance (d_010_) of PffBT4T-2OD films is slightly smaller than that of PffBT4T-2DT films (0.356 nm vs. 0.357 nm), and a slightly larger CCL of π-π stacking (CCL_010_) is exhibited for PffBT4T-2OD films (6.59 nm vs. 6.53 nm), indicating that alkyl side chain length has minimal effect on the interaction between the π-orbitals of the backbones. This is primarily because the small difference in the alkyl side chain length between the two copolymers barely affects the π-π interactions between adjacent molecules. However, Figure 2c shows a sharper (100) peak for PffBT4T-2DT, which has a longer alkyl side chain. As calculated from the FWHM of the (100) peak, the CCL_100_ increases from 30.17 nm on the PffBT4T-2OD films to 37.27 nm on the PffBT4T-2DT films. This indicates that the PffBT4T-2DT films have larger crystal domains and a higher volume fraction of ordered structures compared to PffBT4T-2OD films, which is consistent with the UV-visible spectra results. Higher crystallinity observed in the polymer with longer alkyl side chains may be attributed to the enhanced solubility due to increased polymer–solvent interactions (shown in Note 1 and Figure S2 of the Supporting Information), which facilitates more ordered structures during solvent removal [51]. It should be pointed out that the lamellar stacking distance (d_100_) of PffBT4T-2DT (2.47 nm) is slightly larger than that of PffBT4T-2OD (2.23 nm) due to the steric hindrance caused by the longer alkyl side chain.

### 3.2. Electrical Performance of the SiO_2_-Gated and Ion-Gel-Gated OFETs

To explore the effect of the alkyl side chain length on the carrier transport properties of D-A copolymers, OFET devices with SiO_2_ as the dielectric layer (SiO_2_-gated OFETs) are fabricated in a bottom-gate/bottom-contact (BG/BC) structure, as shown in the inset of Figure S4a. The fabrication process of the SiO_2_-gated OFETs and the characterization of carrier transport are detailed in Note 3 of the Supporting Information. Figure S3a displays the typical transfer curves of BG/BC devices (W/L = 2 mm/20 μm) for PffBT4T-2OD and PffBT4T-2DT films, while the corresponding output curves are shown in Figure S4. The channel current (I_D_) of the PffBT4T-2DT film is found to be approximately 3.3 times higher than that of the PffBT4T-2OD film (3.1 × 10^−^⁴ A vs. 9.5 × 10^−^⁵ A at V_G_ = −50 V) in SiO_2_-gated OFETs. The PffBT4T-2DT film exhibits a high mobility of 0.23 cm^2^/V·s in the saturation regime, representing a 2.4-fold enhancement compared to the PffBT4T-2OD film (0.096 cm^2^/V·s). Such promotion of carrier transport should be correlated with the higher crystallinity and larger crystal domains in the films of PffBT4T-2DT, which possesses longer alkyl side chains than PffBT4T-2OD. Furthermore, Figure S3b shows the variation in hole mobility of the SiO_2_-gated OFETs with channel length, revealing that the mobility difference between the PffBT4T-2OD and PffBT4T-2DT films increases with channel length. PffBT4T-2DT devices exhibit a remarkable enhancement of mobility, while the PffBT4T-2DT devices show a decrease in carrier mobility with an increase in channel length. This is primarily attributed to the stronger interchain interaction and larger crystal domains of PffBT4T-2DT, which results in a more efficient carrier transport pathway in the FET channel compared to PffBT4T-2OD.

**Figure 3:**
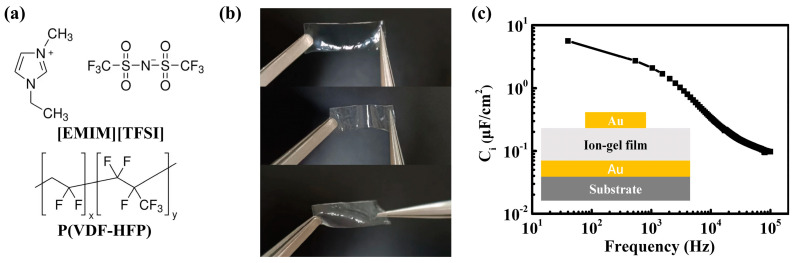
(**a**) The molecular structure of [EMIM]^+^[TFSI]^−^ and P(VDF-HFP). (**b**) The photograph of prepared ion-gel films. (**c**) The specific capacitance–frequency curve of ion-gel film. The inset shows the schematic of an Au/ion-gel/Au capacitor structure.

Ion-gel films were prepared to enhance the electrical performance and enable low voltage operation for OFETs, thanks to their ultrahigh capacitance, making them suitable for applications like electronic skin (e-skin). Figure 3a shows the molecular structure of the ionic liquid containing the cation [EMIM]^+^ and anion [TFSI]^−^, along with P(VDF-HFP) as a flexible polymer matrix in ion-gel films. Figure 3b shows the photographs of ion-gel films in stretched, bent, and twisted states, demonstrating their excellent mechanical flexibility, which is attributed to the low tensile strength of P(VDF-HFP) [52,53]. This also suggests that the ion-gel films may adhere tightly to the polymer films when used as a dielectric layer. A sandwich-type metal–dielectric–metal structure (with an effective area of 0.04 cm^2^), as shown in the inset of Figure 3c, was fabricated to examine the frequency-dependent capacitance of the ion-gel films via the impedance analyzer. As shown in Figure 3c, the ion-gel film exhibits an ultrahigh specific capacitance of 5.6 μF/cm^2^ at 40 Hz, attributed to the formation of an interfacial electric double layer (EDL) [24,25,54], which will allow for a dramatically higher charge density at the ion-gel/semiconductor interface compared to SiO_2_ (15 nF/cm^2^ for 230 nm SiO_2_). For a consistency check, the ion-gel capacitor was also measured using EIS. Figure S5 displays the specific areal capacitance and phase angle of ion-gel film as well as the imaginary part of the specific capacitance as a function of frequency, giving consistent results of the capacitance with those in Figure 3c. The ion-gel film exhibits a specific capacitance of 6.6 μF/cm^2^ and a phase angle of −72.3° at 10 Hz, indicating an almost ideal capacitor.

**Figure 4:**
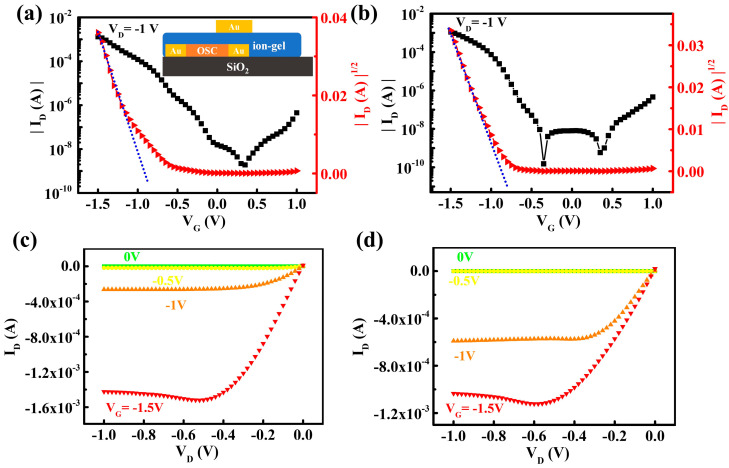
(**a**,**b**) Typical transfer curves of the ion-gel-gated OFETs (W = 2 mm/L = 50 μm) based on PffBT4T-2OD films (**a**) and PffBT4T-2DT films (**b**). The inset of (**a**) illustrates the schematic of ion-gel-gated OFETs on the TG/BC structure. (**c**,**d**) Corresponding output curves of the ion-gel-gated OFETs of PffBT4T-2OD films (**c**) and PffBT4T-2DT films (**d**), respectively.

Ion-gel-gated OFETs employing a top-gated/bottom-contact (TG/BC) structure, as shown in the inset of Figure 4a, were fabricated to explore the effect of the ion-gel dielectric layer on the electrical performance of OFETs. Figure 4a,b present typical transfer curves in the saturation region of the ion-gel-gated OFETs, using PffBT4T-2OD and PffBT4T-2DT films as the active layers, respectively. The OFETs exhibit extraordinarily high drain currents (I_D_) of 1.3 × 10^−3^ A and 1.1 × 10^−3^ A at a low V_G_ of −1.5 V, as well as high I_on_/I_off_ ratios of 7.6 × 10^5^ and 1.5 × 10^6^ for PffBT4T-2OD and PffBT4T-2DT films, respectively. This demonstrates that the large-capacitance ion-gel dielectric enables a significantly high carrier density (9.2 × 10^13^/cm^2^) induced at the semiconductor/dielectric interface, which also remarkably lowers the operating voltage of OFETs. Figure 4c,d show the output curves of the ion-gel-gated OFETs, demonstrating a typical p-type operation. In the slope of the square root of I_D_ versus V_G_ curves in Figure 4a,b, hole mobility of the ion-gel-gated OFETs based on the PffBT4T-2OD and PffBT4T-2DT films is calculated to be 17.7 cm^2^/V·s and 14.5 cm^2^/V·s, respectively, by employing the estimated capacitance value of the ion-gel at 10 Hz in Figure S6 (7.9 μF/cm^2^). It is among the highest values of mobility ever reported for electrolyte-gated (including ion-gel-gated) OFETs [27,28,29,30,40]. Figure S7 presents the variation of mobility and I_on_/I_off_ ratios as a function of channel length in the ion-gated OFETs. Exceptionally high hole mobility and I_on_/I_off_ ratios are observed for both kinds of devices at a variety of channel lengths between 50 and 200 μm, despite that a slight decay in device performance is exhibited with the increased channel length. The excellent performance of these ion-gel OFETs can be attributed to the high film crystallinity and outstanding charge transport capability of PffBT4T-based D-A copolymers and, more importantly, to the full filling of carrier traps via high-density holes induced by the ion-gel. However, it is noteworthy that ion-gated OFETs based on the PffBT4T-2OD films, which exhibit lower crystallinity, exhibit higher electrical performance than those of the PffBT4T-2DT films, in contrast to the behavior observed on the SiO_2_-gated OFETs. The underlying reasons for this discrepancy will be discussed in the subsequent sections. In addition, the transconductance (*g*_m_) of the OFETs is calculated using the following equation:

$$g_{m}=\frac{\partial I_{D}}{\partial V_{G}}$$

This metric is used to evaluate the current driving capability of the ion-gel OFETs. As illustrated in Figure S8, the ion-gel OFETs of the PffBT4T-2OD films also demonstrate superior *g*_m_ in the saturation region compared to the PffBT4T-2DT devices (4.5 mS vs. 3.4 mS), indicating higher capability for the applications such as the active matrix driving arrays.

**Figure 5:**
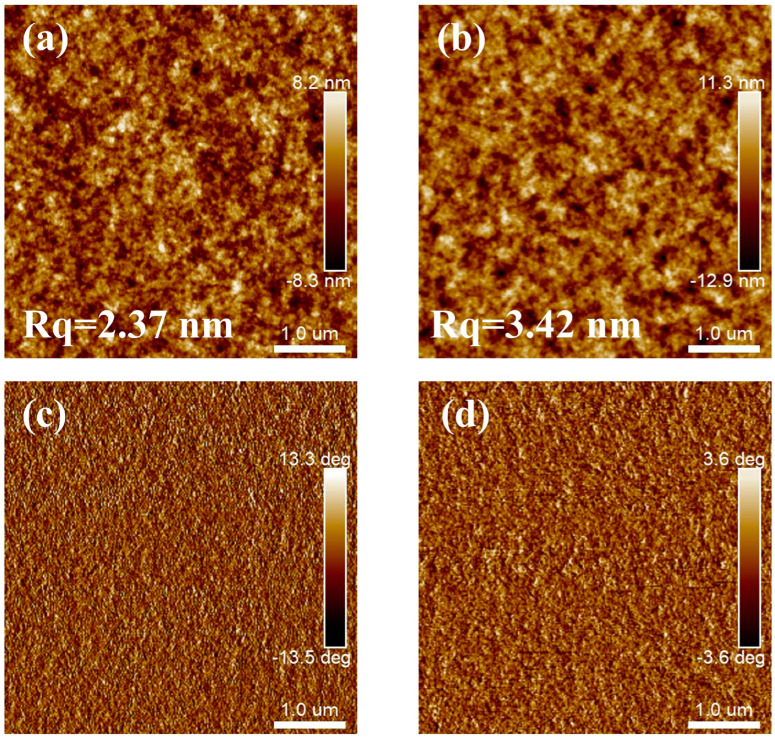
AFM height images (**a**,**b**) and phase images (**c**,**d**) of PffBT4T-2OD films (**a**,**c**) and PffBT4T-2DT films (**b**,**d**) in tapping mode.

The surface morphology of the PffBT4T-2OD and PffBT4T-2DT films is characterized using tapping-mode AFM, as shown in Figure 5. The AFM height images in Figure 5a,b reveal a higher root-mean-square roughness (Rq) on the PffBT4T-2DT films compared to the PffBT4T-2OD films (3.42 nm vs. 2.37 nm), despite the fact that both of the films consist of a tiny nanofibril-like structure. The increased surface roughness of the PffBT4T-2DT films can be attributed to their higher crystallinity and larger crystal domains [55,56]. It is proposed that the superior performance of ion-gel-gated OFETs based on PffBT4T-2OD films is attributed to the smoother surface, facilitating better contact between the ion-gel films and the semiconductor layer. In addition, the shorter alkyl side chain of PffBT4T-2OD allows greater ion permeability, which is favorable for channel conductivity and ionic–electronic coupling in ion-gel-gated OFETs [57,58]. Moreover, static contact angle measurements were applied to investigate the surface properties of PffBT4T-2OD and PffBT4T-2DT films. As shown in Figure S9, PffBT4T-2OD films demonstrate greater hydrophilicity, likely due to their shorter alkyl side chains [59], which is beneficial to the adhesion of the ion-gel dielectric to the polymer films. Therefore, although the electrical performance of ion-gated OFETs is strongly related to the crystallinity and molecular ordering of polymer films [30], the interface between the ion-gel films and polymer films, along with the surface morphology and interfacial interaction, plays a more critical role in the performance of ion-gel-gated transistors [60,61].

### 3.3. Electrical Performance of Flexible Ion-Gel-Gated OFETs

**Figure 6:**
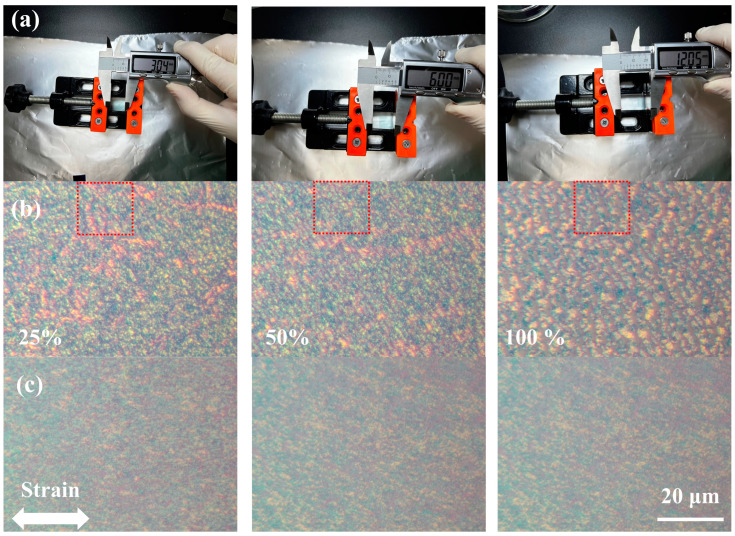
(**a**) Photograph illustration of stretching polymer films at the strain of 25%, 50%, and 100%. (**b**,**c**) OM images of the stretched PffBT4T-2OD films (**b**) and blended films (PffBT4T-2DT/SEBS = 7:3) (**c**) under various strains, where the white arrow denotes the strain direction.

To achieve high flexibility of ion-gel-gated OFETs, a blend of PffBT4T-2OD and the insulating elastomer SEBS [62,63] (7:3 in weight ratio) was used as the semiconducting layer. To explore the mechanical properties of the PffBT4T-2OD/SEBS blend films, the blend films spun on the octadecyltrichlorosilane (OTS)-modified SiO_2_/Si substrates were transferred onto a PDMS stamp via a soft-contact lamination method and subjected to various tensile strains, as shown in Figure 6a. After stretching, the films were laminated again onto the SiO_2_/Si substrates for morphological characterization. Optical microscope (OM) images of pristine PffBT4T-2OD films and PffBT4T-2OD/SEBS (7:3) blend films under various strains are shown in Figure 6b,c. As shown in Figure 6b, numerous micro-cracks perpendicular to the stretching direction appeared in the PffBT4T-2OD film after 25% strain. Both the size and number of cracks increased significantly with the increase in strain. However, for the PffBT4T-2OD/SEBS blend films (in Figure 6c), no micro-cracks were observed at even 100% tensile strain, indicating a crack onset strain exceeding 100%. The high stretchability of the blend films should be attributed to efficient stress dissipation provided by the flexible insulating polymer SEBS.

**Figure 7:**
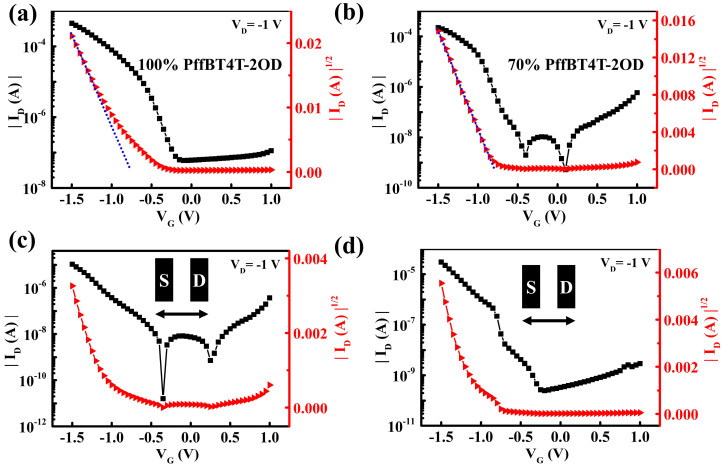
Typical transfer curves of the ion-gated OFETs based on pure PffBT4T-2OD films (**a**,**c**) and PffBT4T-2OD/SEBS (7:3) blend films (**b**,**d**) before tensile strain (**a**,**b**) and under the strain of 100% (**c**,**d**), respectively. The strain direction is parallel to the direction of channel current. The channel length (L) and channel width (W) are 200 μm and 2 mm, respectively.

Figure 7a shows transfer curves of the ion-gel-gated OFETs based on pristine PffBT4T-2OD films, which exhibit a hole mobility of 13.06 cm^2^/V·s at a channel length of 200 μm. In contrast, for the ion-gel-gated OFETs employing the PffBT4T-2OD/SEBS (7:3) blend films (in Figure 7b), a hole mobility of 8.6 cm^2^/V·s is achieved, indicating that PffBT4T-2OD maintains favorable charge transport properties even if a large amount of insulating SEBS is incorporated into the film. This should be due to the uniform phase-separated structure in the hybrid film in which the nano-domains of PffBT4T-OD are homogeneously distributed in the SEBS matrix (as shown in Figure S10 of the Supporting Information) to enable the formation of an intimate semiconductor/ion-gel interface. Ion-gel-gated OFETs, based on pure PffBT4T-2OD films and PffBT4T-2OD/SEBS (7:3) blend films under 100% tensile strain, were also fabricated to investigate the effect of tensile strain on carrier transport of the blend-film devices. Figure 7c shows the transfer curve of an ion-gel-gated OFET using the PffBT4T-2OD film subjected to 100% tensile strain, with the channel current parallel to the tensile direction. The device exhibits a low mobility of only 0.875 cm^2^/V·s, representing a 94% reduction in mobility compared to the unstretched film. This significant reduction is mainly due to the formation of many micro-cracks perpendicular to the tensile direction in the PffBT4T-2OD film, which severely obstructs charge transport pathways along the channel current. However, for the strained PffBT4T-2OD/SEBS blend film, as shown in Figure 7d, the ion-gel-gated OFET exhibits a hole mobility of 3.75 cm^2^/V·s, retaining 43.6% of its original mobility in the unstretched state. The lowered strain sensitivity of charge transport properties for the PffBT4T-2OD/SEBS film is correlated with a minimal change in the microstructure (e.g., remarkably suppressed formation of micro-cracks) of the blend films under tensile strain. The characteristics of low operation voltage, high stretchability, and superior mobility offered by the ion-gel-gated OFETs of the PffBT4T-2OD-based blend films present a promising capability for high-performance wearable electronics applications.

## 4. Conclusions

This study demonstrates that the length of alkyl side chains is crucial in determining both the electrical performance and mechanical flexibility of ion-gel-gated OFETs. While longer alkyl side chains in PffBT4T-2DT enhance crystallinity and charge mobility in SiO_2_-gated OFETs, the shorter side chains of PffBT4T-2OD offer a smoother surface and stronger interaction with the ion-gel dielectric, leading to superior performance in ion-gel-gated OFETs. PffBT4T-2OD, when blended with SEBS, also demonstrates excellent mechanical flexibility, retaining 45% of its initial mobility under 100% strain. These findings emphasize the crucial need to balance molecular packing with semiconductor–dielectric interactions in order to optimize both electrical performance and flexibility in ion-gel-gated OFETs. This work holds significant implications for the development of wearable and stretchable electronics, where both high performance and mechanical durability are essential.

## Figures and Tables

**Table 1:** Molecular stacking parameters of PffBT4T-2OD and PffBT4T-2DT films.

	π-π Stacking (010)				Lamellar Stacking (100)			
	*q* (Å^−1^)	d_010_ (nm)	FWHM (°)	CCL_010_ (nm)	*q* (Å^−1^)	d_100_ (nm)	FWHM (°)	CCL_100_ (nm)
PffBT4T-2OD	1.763	0.356	1.02	6.59	0.282	2.23	0.21	30.17
PffBT4T-2DT	1.760	0.357	1.03	6.53	0.254	2.47	0.17	37.27

## Data Availability

Derived data supporting the findings of this study are available from the corresponding author upon reasonable request due to privacy.

## Supplementary Materials

The following supporting information can be downloaded at: https://www.mdpi.com/article/10.3390/polym16233287/s1, Figure S1: the normalized UV-visible absorption spectra of PffBT4T-2OD and PffBT4T-2DT solutions. Figure S2: (a,b) the profiles of the (010) diffraction peak (a) and the (100) diffraction peak (b) for both the PffBT4T-2OD and PffBT4T-2DT films shown in Figure 2c and Figure 2d, respectively. Figure S3: (a) Typical transfer curves of the SiO_2_-gated OFETs based on spin-coated PffBT4T-2OD and PffBT4T-2DT films. (b) Variation of the extracted hole mobility with a function of channel length. Figure S4: (a,b) Typical output curves of the SiO_2_ gated OFETs based on PffBT4T-2OD films (a) and PffBT4T-2DT films (b). The inset of Figure S4a shows the illustration of a SiO_2_-based OFET in the BG/BC structure. Figure S5: (a) The specific areal capacitance and phase angle versus frequency; (b) The imaginary part of the specific capacitance (C’’) versus frequency. Figure S6: The specific capacitance–frequency curve of an ion-gel-based capacitor. The dashed line (blue) denotes the extrapolation of the C-f curve at a low frequency (lower than 40 Hz). A capacitance value of 7.9 μF/cm^2^ can be estimated at the frequency of 10 Hz, which is consistent with the result (6.6 μF/cm^2^) measured from the EIS in Figure S5a. The value of Ci at 10 Hz is employed to calculate carrier mobility of the ion-gel-gated OFETs since the frequency (10 Hz) roughly corresponds to the gate-sweeping rate (0.2 V/s) in the measurements of transfer characteristics. Figure S7: (a,b) The channel length dependence of hole mobility and I_on_/I_off_ ratios extracted from ion-gel-gated OFETs based on PffBT4T-2OD films (a) and PffBT4T-2DT films (b), respectively. Figure S8: (a,b) The V_G_ dependence of transconductance (*g*_m_) of the ion-gel-gated OFETs based on the PffBT4T-2OD film and PffBT4T-2DT film, extracted from the transfer curves (V_D_ = −1 V) in Figure 4a,b in the main text. Figure S9: Static water contact angle (a,c) and diiodomethane contact angle (b, d) of PffBT4T-2OD films (a,b) and PffBT4T-2DT films (c,d). Figure S10: AFM height images (a) and phase images (b) of PffBT4T-2OD/SEBS (7:3) blended films in the tapping mode. References [64,65] are cited in the supplementary materials.
