# Supplementary material for: Effect of Alkyl Side Chain Length on Electrical Performance of Ion-Gel-Gated OFETs Based on Difluorobenzothiadiazole-Based D-A Copolymers
Source: Polymers (Basel). 2024 Nov 26;16(23):3287. doi: 10.3390/polym16233287 (PMC11644371; doi:10.3390/polym16233287)
Supplement: Supplementary file 1 [file polymers-16-03287-s001.zip › polymers-3316483-supplementary.pdf]

Effect of Alkyl Side Chain Length on Electrical Performance of Ion-Gel-Gated OFETs Based on Difluorobenzothiadiazole-Based D-A Copolymers

Han Zhou [1,2], Zaitian Cheng [1,2], Guoxing Pan [1,3,*], Lin Hu [1] and Fapei Zhang [1,*]

[1] Anhui Key Laboratory of Low-Energy Quantum Materials and Devices, High Magnetic Field Laboratory, HFIPS, Chinese Academy of Sciences, Hefei 230031, China
[2] Science lsland Branch, Graduate School, University of Science and Technology of China, Hefei 230026, China
[3] Institutes of Physical Science and Information Technology, Anhui University, Hefei 230601, China
* Correspondence: panguoxing@ahu.edu.cn (G.P.); fzhang@hmfl.ac.cn (F.Z.)

Note 1. UV-visible spectra of the PffBT4T-polymers solutions

Figure S1 shows the UV-visible specta of the PffBT4T-polymers dissloved in o-DCB (0.05 mg/ml). Two strong absorption bands are observed in the ranges of 300–500 nm and 500–750 nm, indicating strong interchain aggregation even in the solution state for both the polymers. The high-energy band (0-1 peak) is attributed to the π–π* excitation while the low-energy band (0-0 peak) can be assigned to the intramolecular charge transfer transition [1]. However, the ratio of the $A_{0\text{-}0}/A_{0\text{-}1}$ is 1.08 and 0.81 for the PffBT4T-2OD and PffBT4T-2DT solutions respectively, revealing a stronger interaction between PffBT4T-2DT and o-DCB molecules.

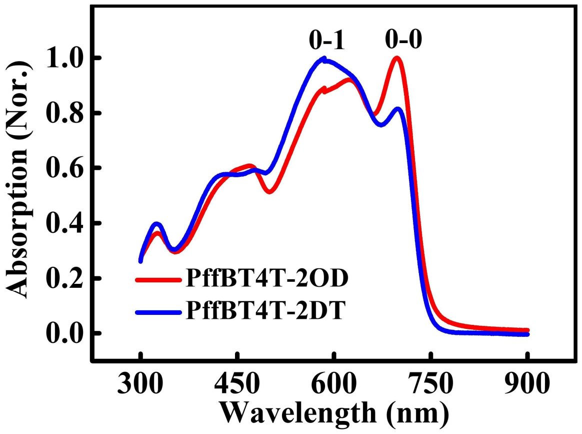

Figure S1 The normalized UV-visible absorption spectra of the PffBT4T-2OD and PffBT4T-2DT solutions.

Note 2. Calculation of crystalline correlation length (CCL) and d-spacing

The crystalline correlation length (CCL) is estimated from the full width at half maximum (FWHM) and the diffraction peak position, based on Scherrer equation:

$$D = \frac{k\lambda}{\beta cos\theta}$$

where D is the crystallite size, λ is the X-ray wavelength in our experiment (0.124 nm), θ is the Bragg angle of the diffraction peak (in radians), and β is the FWHM of a diffraction peak in radians.

The π-π stacking distance (**d_{010}**) and the lamellar stacking distance (**d_{100}**) is calculated from the position of the (010) and (100) peak respectively, based on Bragg diffraction formula ($d = \frac{2\pi}{q}$, where **q** is the wavevector at the (010) and (100) peak position).

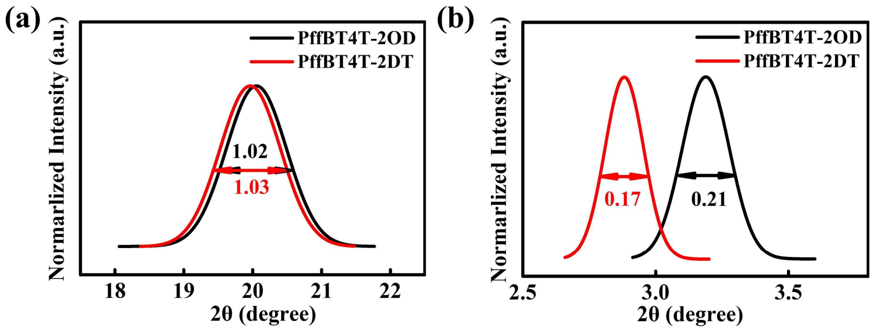

Figure S2 (a-b) The profiles of the (010) diffraction peak (a) and the (100) diffraction peak (b) for both the PffBT4T-2OD and PffBT4T-2DT films shown in Figure 2c and Figure 2d, respectively.

Note 3. Fabrication and electrical properties characterization of SiO2-based OFETs

PffBT4T-2OD and PffBT4T-2DT were dissolved in o-dichlorobenzene. The solutions (6 mg/mL) were stirred at 80°C for 12 hours in a nitrogen (N_2) glove box to ensure complete dissolution. The dissolved PffBT4T-2OD and PffBT4T-2DT solutions were then spin-coated onto clean SiO_2/n++ Si substrates pre-patterned with the ITO/Au (10 nm/30 nm) source/drain electrode arrays by photolithography. Then the films were annealed at 140 °C for 1h under the atomsphere of N_2, to finish the fabrication of a bottom-gate/bottom-contact (BG/BC) FET device. The elecetrode arrays define the channel widths (W) of 2.0 mm and different channel length (L) of 2.5 μm, 5 μm, 10 μm, and 20 μm for the SiO_2-gated OFETs, respectively. The electrical characteristics of the devices were measured using a Keithley 2612A source meter on a probe station in a nitrogen atmosphere. The field-effect mobility was calculated from the slope of the square root of the drain current (I_D) versus gate voltage (V_G) curves in the saturation regime, based on the standard transistor equation:

$$I_D = \frac{W}{2L}\mu C_i(V_G - V_T)^2$$

Where C_i is the area capacitance of the dielectric (15 nF/cm^2 for 230-nm SiO_2), V_T is threshold voltage.

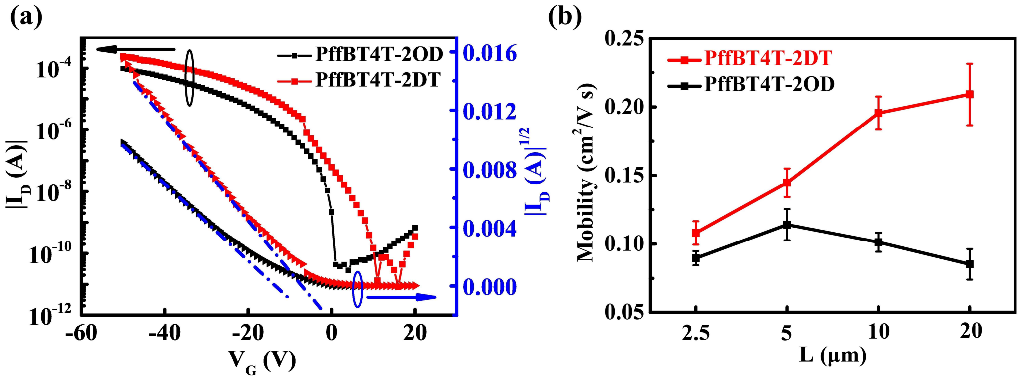

Figure S3 (a)Typical transfer curves of the SiO_2 gated OFETs based on the spin-coated PffBT4T-2OD and PffBT4T-2DT films. (b) Variation of the extracted hole mobility with a function of channel length.

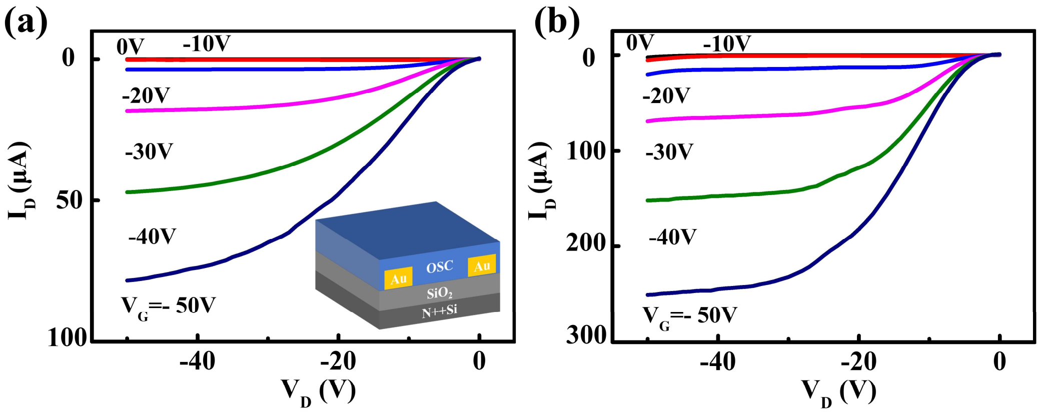

Figure S4 (a-b)Typical output curves of the SiO_2 gated OFETs based on the PffBT4T-2OD films (a) and the PffBT4T-2DT films (b). The inset of Figure S4a shows the illustration of a SiO_2-based OFET in the BG/BC structure.

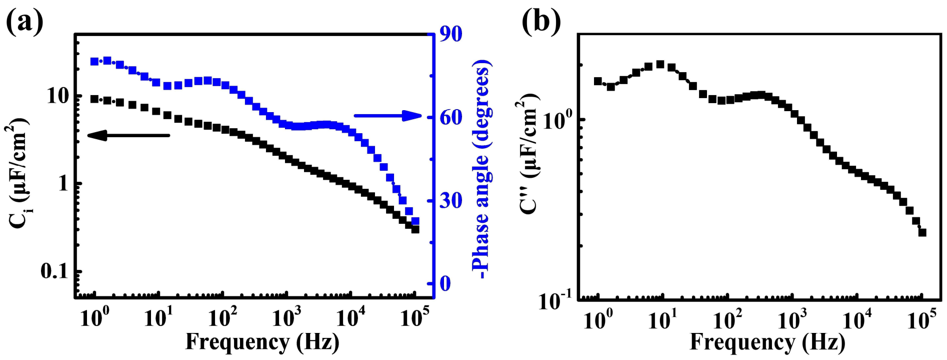

Figure S5 (a) The specific areal capacitance and phase angle versus frequency; (b) The imaginary part of the specific capacitance (C″) versus frequency.

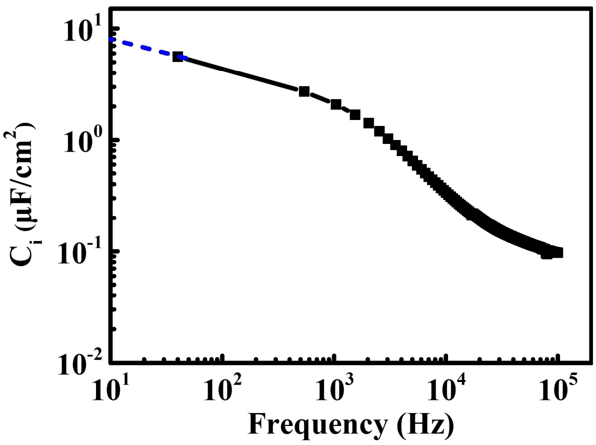

Figure S6 The specific capacitance-frequency curve of ion-gel based capacitor. The dashed line (blue) denotes the extrapolation of the C-f curve at low frequency (lower than 40 Hz). A capacitance value of 7.9 μF/cm^2 can be estimated at the freqenucy of 10 Hz, which is well consistent with the result (6.6 μF/cm^2) measured from the EIS in Figure S5a. The value of C_i at 10 Hz is employed to calculate carrier mobility of the ion-gel gated OFETs, since the frequency (10 Hz) roughly corresponds to the gate-sweeping rate (0.2 V/s) in the measurements of transfer characteristics

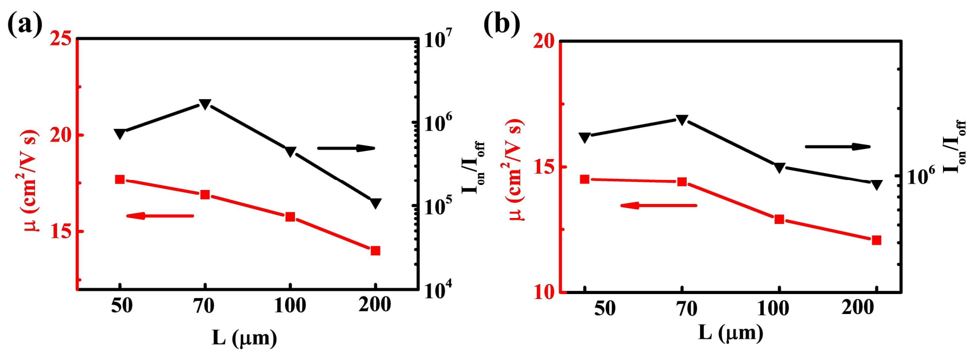

Figure S7 (a-b) The channel-length dependence of hole mobility and I_{on}/I_{off} ratios extracted from ion-gel gated OFETs based on the PffBT4T-2OD films (a) and the PffBT4T-2DT films (b), respectively.

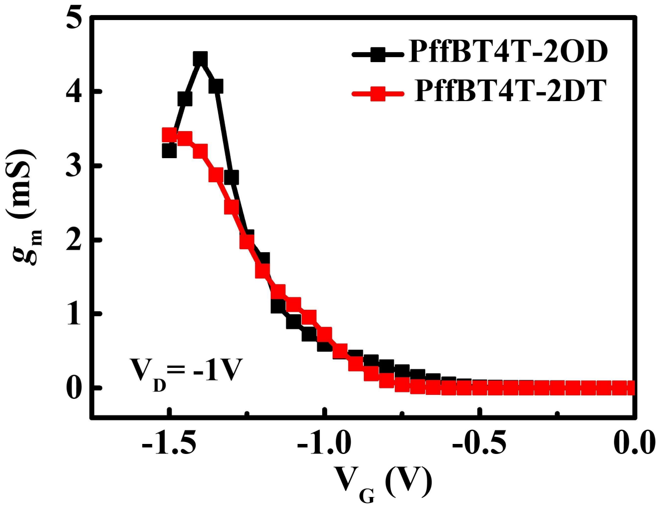

Figure S8 (a-b) The V_G dependence of transconductance (g_m) of the ion-gel gated OFETs based on the PffBT4T-2OD film and PffBT4T-2DT film, extracted from the transfer curves (V_D= -1 V) in Figure 4a-b in main text.

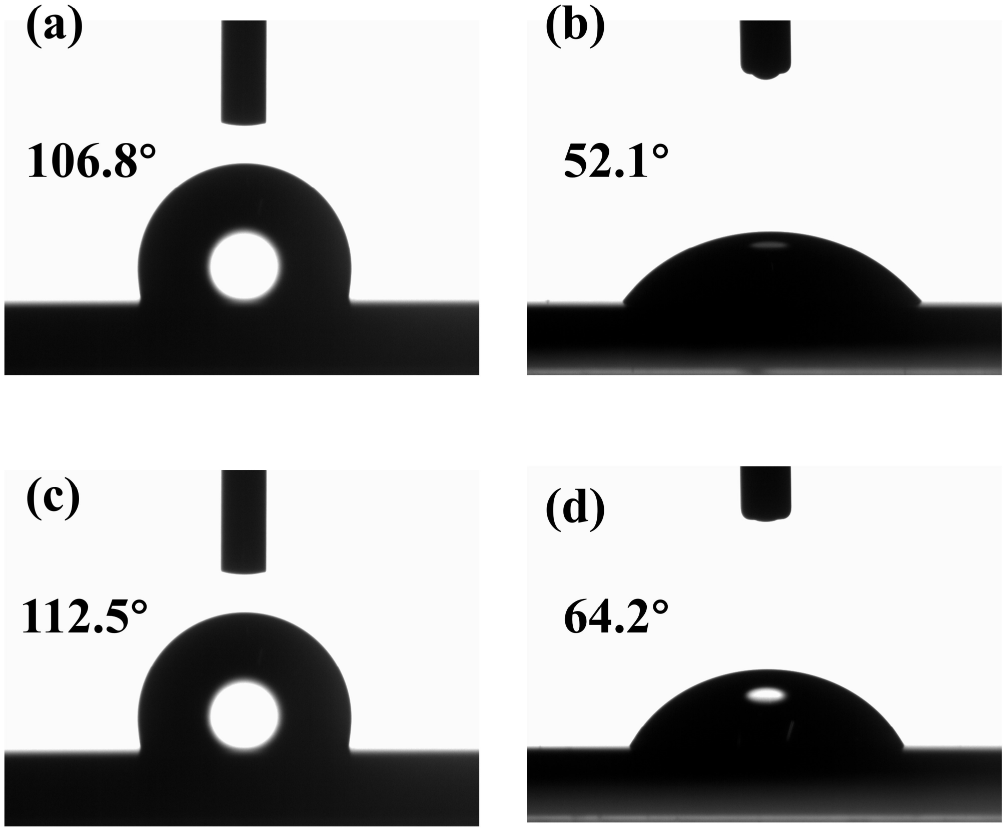

Figure S9 Static water contact angle(a, c) and diiodomethane contact angle (b, d) of PffBT4T-2OD films (a-b) and PffBT4T-2DT films (c-d).

Note 4 The morphology of PffBT4T-2OD/SEBS (7:3) blended films

The AFM phase images of the PffBT4T-2OD/SEBS (7:3) blend films are shown in Figure S7b, where the white nano-islands on the film surface are primarily SEBS nanoclusters [2-4]. It can be seen that the SEBS nanoclusters are uniformly distributed on the blended film's surface, indicating the homogeneous integration of PffBT4T-2OD crystalline domains and SEBS throughout the blend. The uniform phase-separated structure on the surface of the blended films not only provides the continuous channels for carrier transport but also ensures ample sites for charge accumulation in ion-gel gated OFETs.

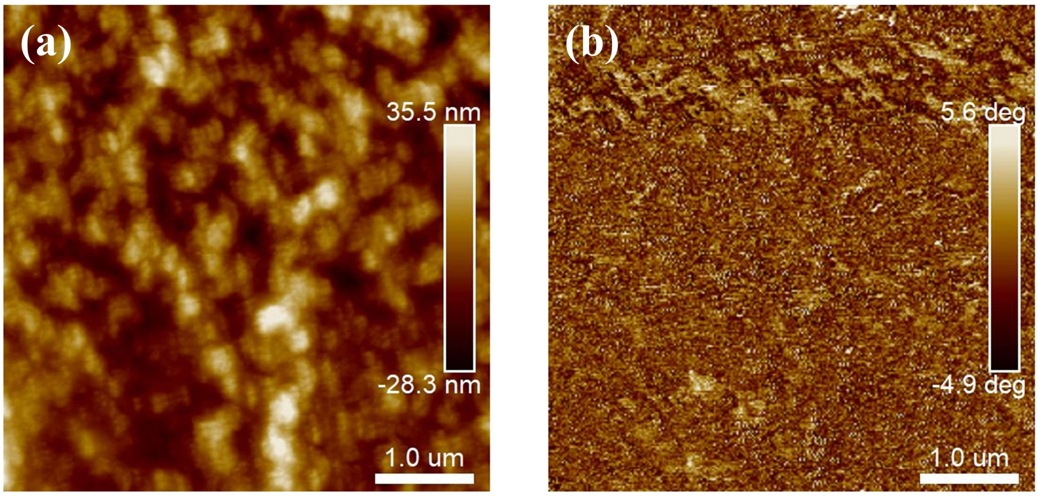

Figure S10 AFM height images (a) and phase images (b) of PffBT4T-2OD/SEBS (7:3) blended films in tapping mode.

References

1. Liu, Y.; Zhao, J.; Li, Z.; Mu, C.; Ma, W.; Hu, H.; Jiang, K.; Lin, H.; Ade, H.; Yan, H. Aggregation and morphology control enables multiple cases of high-efficiency polymer solar cells. *Nat. Commun.* **2014**, *5*, 5293.
2. Zhang, T.; Liu, Y.; Zhang, L.; Wang, S.; Li, J.; Zuo, J.; Yu, X.; Zhang, Q.; Han, Y. Constructing a desired nanofibril network morphology for stretchable polymer films by weakening the intermolecular interaction of a conjugated polymer in an elastomer matrix and extending the film-forming time. *J. Mater. Chem. C* **2023**, *11*, 2302-2315.
3. Guo, S.; Tong, Y.; Wang, X.; Zhang, M.; Yu, H.; Ren, H.; Tang, Q.; Lu, G.; Liu, Y. Brittle PCDTPT based elastic hybrid networks for transparent stretchable Skin-Like electronics. *Advanced Electronic Materials* **2023**, *9*, 2200438.
4. Lei, Y.; Deng, P.; Zhang, Q.; Xiong, Z.; Li, Q.; Mai, J.; Lu, X.; Zhu, X.; Ong, B.S. Hydrocarbons-Driven crystallization of polymer semiconductors for Low-Temperature fabrication of High-Performance organic Field-Effect transistors. *Adv. Funct. Mater.* **2018**, *28*, 1706372.
